# Convergent Adaptation to Quantitative Host Resistance in a Major Plant Pathogen

**DOI:** 10.1128/mBio.03129-20

**Published:** 2021-02-23

**Authors:** Jean Carlier, François Bonnot, Véronique Roussel, Sébastien Ravel, Reina Teresa Martinez, Luis Perez-Vicente, Catherine Abadie, Stephen Wright

**Affiliations:** a CIRAD, UMR PHIM, Montpellier, France; b PHIM, Plant Health Institute, Univ Montpellier, INRAe, CIRAD, Institut Agro, Montpellier, France; c IDIAF, Santo Domingo, Dominican Republic; d INISAV, Havana, Cuba; e CIRAD, UMR PHIM, Capesterre-Belle-Eau, Guadeloupe, France; f University of Toronto, Toronto, Ontario, Canada; Cornell University

**Keywords:** convergent adaptation, fungal plant pathogen, genome scan, *Musa*, quantitative pathogenicity, quantitative resistance, pool-seq, *Pseudocercospora fijiensis*

## Abstract

Plant pathogens can adapt to quantitative resistance, eroding its effectiveness. The aim of this work was to reveal the genomic basis of adaptation to such a resistance in populations of the fungus Pseudocercospora fijiensis, a major devastating pathogen of banana, by studying convergent adaptation on different cultivars. Samples from *P. fijiensis* populations showing a local adaptation pattern on new banana hybrids with quantitative resistance were compared, based on a genome scan approach, with samples from traditional and more susceptible cultivars in Cuba and the Dominican Republic. Whole-genome sequencing of pools of *P. fijiensis* isolates (pool-seq) sampled from three locations per country was conducted according to a paired population design. The findings of different combined analyses highly supported the existence of convergent adaptation on the study cultivars between locations within but not between countries. Five to six genomic regions involved in this adaptation were detected in each country. An annotation analysis and available biological data supported the hypothesis that some genes within the detected genomic regions may play a role in quantitative pathogenicity, including gene regulation. The results suggested that the genetic basis of fungal adaptation to quantitative plant resistance is at least oligogenic, while highlighting the existence of specific host-pathogen interactions for this kind of resistance.

## INTRODUCTION

When infection is possible, interactions between plants and pathogens can be characterized by several quantitative traits related to disease development (reviewed in references 1 and 2). Overall, the values arising from these quantitative traits result from the pathogen effect (quantitative pathogenicity, often called aggressiveness in the plant pathology literature), host effect (quantitative resistance [QR]), and their interaction. Quantitative resistance (QR) is generally thought to be durable and is therefore being used to an increasing extent in crop protection (3). QR can be distinguished from so-called effector-triggered resistance (ETR), or immunity (ETI), in which major genes confer near-complete protection after recognition of effectors produced by some pathogen genotypes that are often referred to as avirulent genotypes, as reviewed in reference 4. Many ETR genes with large effects are not durable because of their specificity to pathogen genotypes, and it is generally thought that this is not the case for QR. It has been demonstrated, however, that this type of resistance might be eroded following pathogen adaptation and that some level of specificity may exist (reviewed in references 3, 5, and 6). Furthermore, some QR is caused by ETR genes that are partially effective against certain pathogen genotypes (5). The durability of quantitative host resistance has recently been questioned, and there is a need for more information on the genetic basis of quantitative pathogenicity in pathogens and on mechanisms underlying its evolution.

The genetic basis of quantitative pathogenicity in plant pathogens has received relatively little attention, in contrast with studies dealing with the QR of their plant hosts (7). Functional studies of genes involved in pathogenicity have mostly been focused on so-called effectors, which are secreted molecules that modulate pathogen-host interactions (8, 9). Effectors were initially characterized as small secreted proteins (SSPs) rich in cysteine (10). Based on these characteristics and the ability to sequence fungal genomes, *in silico* analysis has greatly extended the list of effector candidates, but *in silico* analysis may overlook some of them if they are not SSPs. New predictors based on machine learning have been developed recently to improve predictions of fungal effectors (11), but these predictions could be too stringent or include false positives, depending on the strategy used. Furthermore, other kinds of genes, such as those involved in gene regulation, may play an important role in quantitative pathogenicity, and functional studies generate little information on gene evolution. Thus, complementary approaches other than functional analyses are needed to gain further insight into the genetic basis of quantitative pathogenicity and how it may evolve during adaptation to QR.

Fungi outnumber other plant pathogens and are responsible for a range of serious plant diseases (12). A first comprehensive quantitative trait locus (QTL) mapping analysis of fungal quantitative pathogenicity was published recently for the wheat pathogen Zymoseptoria tritici (13). A complex genetic architecture was identified, along with large- and small-effect QTLs for all the traits analyzed, as well as some candidate genes, including potential effectors and other gene classes. In addition, population genomics can be used to identify the targets of positive selection, thereby providing a complementary approach to identify genes of importance for pathogenicity. A few population genomics studies based on whole-genome sequencing have been published on plant-pathogenic fungi to identify genomic regions under selection (14–17). However, the sampling designs used in those studies were not focused on adaptation to given hosts since the samples came from various locations or countries and the corresponding pathogen populations undoubtedly had various hosts and coped with other external factors.

Studying convergent adaptive evolution (or convergent adaption) can shed light on the ecological and molecular basis of adaptive traits (18, 19). As in the work of Lee and Coop (19), the term convergence used here covers all cases regarding the repeated evolution of similar traits across independent lineages, without distinguishing between convergent and parallel evolution. According to this definition, adaptive convergence can arise due to changes at independent loci, independent changes at the same loci, and/or identical changes at a locus due to independent mutations or selection on standing genetic variation. Recent studies on a number of organisms have shown signs that convergent adaptation involving the same genes is surprisingly common (18). Population genomics can be used to assess convergent adaption based on a paired design and sampling multiple pairs of populations evolving in contrasted environments which can be viewed as replicates of a natural experiment (20–23). Genomic regions underlying convergent adaptation involving a shared genetic basis can be identified by searching for loci that are strongly differentiated in multiple pairs of populations compared to the genomic background. Such studies can benefit from genome sequencing of pools of isolates (pool-seq)—a cost-effective method that enables allele frequency estimation using large sample sizes (24). Applying this approach to fungal plant pathogens could be helpful for identifying genes involved in quantitative pathogenicity and in adaptation to different host cultivars.

The ascomycete fungus Pseudocercospora fijiensis is responsible for black leaf streak disease (BLSD, also known as black Sigatoka) of banana, which is a major agronomic constraint for that crop (recently reviewed in the work of Guzman et al. [25]). In the last 40 years, this emerging disease has spread from Asia throughout the intertropical banana production zone. It is one of the most marked examples of a recent pandemic in the plant kingdom and is considered to be one of the most serious threats to food security (26). Studies of the population history of *P. fijiensis* revealed that demographic events have accompanied its spread into tropical areas (27). In the Americas, BLSD was first detected in Honduras in 1974 and within a few decades it then spread throughout the Latin America-Caribbean region (25). Some banana varieties featuring QR (derived from breeding programs) have been used in recent years to control this disease in some countries in this region, such as Cuba and the Dominican Republic (DR). However, a local adaptation of pathogen populations eroding this QR has been recently detected based on a paired population design and cross-inoculations on plants (6). This first study did not provide any information on the genetic basis of the local adaptation detected. Due to the existence of regular sexual reproduction in natural *P. fijiensis* populations (27) and the recent publication of a reference genome for this fungus (28), the *P. fijiensis*/banana pathosystem is an appropriate model to be used in population genomics approaches to further characterize adaptation to quantitative host resistance.

In this study, we set out to test for convergent adaptation to the quantitative resistance of banana in populations of the fungus *P. fijiensis* and concurrently to reveal the genomic basis of this adaptation. To this end, we developed a genome scan approach based on pool-seq of *P. fijiensis* samples collected from resistant and susceptible banana cultivars in Cuba and the Dominican Republic based on the same paired population design as that used in the work of Dumartinet et al. (6).

## RESULTS

### Pool-seq and SNP calling.

**FIG 1:**
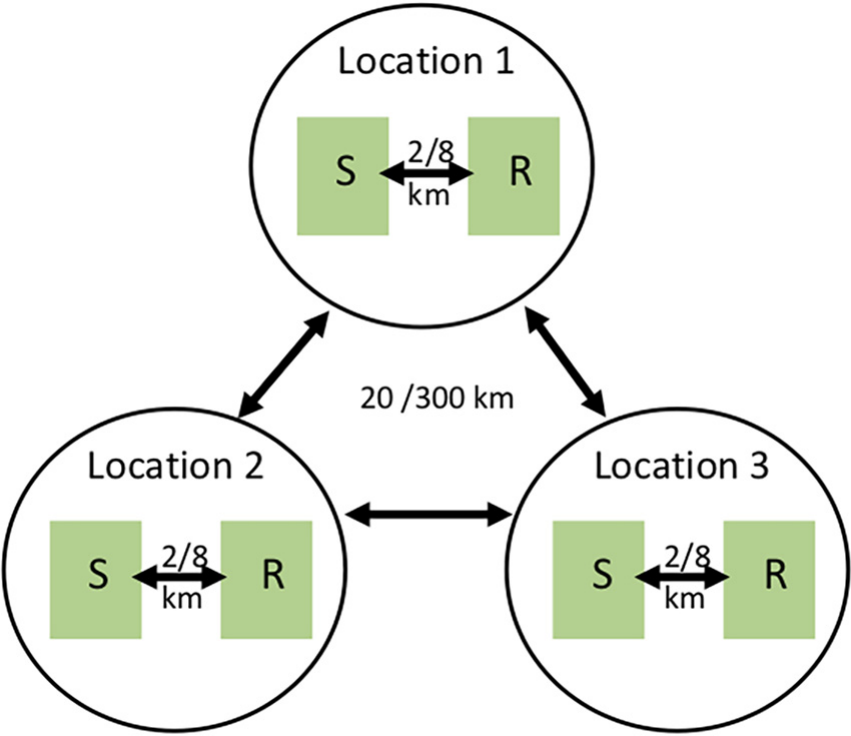
Paired population design for samples collected in the Dominican Republic and Cuba. At each location, about 40 *P. fijiensis* isolates on average were collected from a banana plantation in which a resistant hybrid (R) and a susceptible variety (S) were grown.

Six samples were collected at three locations in 2011 in the Dominican Republic (DR) and in Cuba using a paired population sampling design (Fig. 1 and Table 1). This design, with samples from different population pairs and locations (representing replicates), is a powerful strategy that strengthens the detection of selection in some genomic regions (18, 29, 30). In each country, infected banana leaves were collected at three locations 20 to 300 km apart, and from two banana plantations at each location, i.e., one planted with a susceptible variety and the other with a resistant variety. As the mean dispersal distance of *P. fijiensis* ascospores has been shown to be around a few hundred meters (31), we opted for a distance of around 2 to 8 km between the two plantations of the same location to limit gene flow, which might have counteracted host selection (32). The same varieties were collected at the three locations of each country and could therefore be considered replicates, but the sampled varieties differed between countries. The two susceptible cultivars, i.e., ‘Macho’ and ‘Macho 3/4’ from DR and Cuba, respectively, both belong to the banana AAB genomic group and the plantain subgroup, which is genetically very homogenous (33). The two resistant cultivars, i.e., ‘FHIA 21’ and ‘FHIA 18’ from DR and Cuba, respectively, are tetraploid hybrids (AAAB genomic group) that were both created by the Fundación Hondureña de Investigación Agrícola (FHIA), with a BLSD-resistant diploid hybrid (SH-3142) as common male parent and different BLSD-susceptible triploids as female parent (34, 35). Samples from one location in Honduras—the country where BLSD was first detected in the Latin America-Caribbean zone (27)—were included as reference populations. They were collected on two plots planted with different susceptible varieties (‘French sombre,’ another plantain, and ‘Grande Naine,’ belonging to the AAA genomic group and Cavendish subgroup). On average, 42.25 haploid isolates representing different individuals from each population were pooled and sequenced (pool-seq). The sample size of the pools analyzed was limited by the number of isolates we could obtain from infected plants. These sizes might seem suboptimum as a pool size of 50 diploid individuals is usually recommended for accurate allelic frequency (AF) estimation (24). However, a recent theoretical study showed that the limit of 50 individuals may be overly conservative if some precautions are taken in DNA pooling and sequencing (36), as was the case in this study (see Fig. S1 in the supplemental material). Furthermore, experimental studies comparing AFs between individuals and pool sequencing have shown that satisfactory AF estimates can be obtained with pool sizes of around 20 to 25 (37, 38) and even 12 (39) with appropriate data filtering. After filtering, we identified 576,627 and 753,001 biallelic single nucleotide polymorphisms (SNPs) in the DR and Cuba samples, respectively, and 579,597 when combining all the samples from DR, Cuba, and Honduras.

**TABLE 1:** *Pseudocercospora fijiensis* samples analyzed

Country (code)	Location (code)	Cultivar of origin			Pool-seq size^*a*^	Population code
		Name (code)	Phenotype	Group		
Dominican Republic (DR)	La Vega (20)	Macho (S1)	Susceptible	AAB	42	DR1 S1
		FHIA21 (R1)	Resistant	AAAB	37	DR1 R1
	Moca (20)	Macho (S1)	Susceptible	AAB	37	DR2 S1
		FHIA21 (R1)	Resistant	AAAB	32	DR2 R1
	San Francisco (3)	Macho (S1)	Susceptible	AAB	47	DR3 S1
		FHIA21 (R1)	Resistant	AAAB	41	DR3 R1
Cuba (CU)	Villa Clara (20)	Macho 3/4 (S2)	Susceptible	AAB	40	CU1 S2
		FHIA18 (R2)	Resistant	AAAB	48	CU1 R2
	Ciego de Avila (20)	Macho 3/4 (S2)	Susceptible	AAB	49	CU2 S2
		FHIA18 (R2)	Resistant	AAAB	58	CU2 R2
	Matanzas (3)	Macho 3/4 (S2)	Susceptible	AAB	38	CU3 S2
		FHIA18 (R2)	Resistant	AAAB	38	CU3 R2
Honduras (HN)	La Lima (20)	Grande Naine (S3)	Susceptible	AAA	27	HN1 S3
		French sombre (S4)	Susceptible	AAB	30	HN1 S4

a Number of *P. fijiensis* isolates pooled for sequencing.

10.1128/mBio.03129-20.1 FIG S1 Number of SNPs detected between four sequencing replicates of the same pool (DR3 R2) as a function of the minimum read count (min.rc) and the minimum allele frequency (MAF). Download FIG S1, TIF file, 2.0 MB. Copyright © 2021 Carlier et al. 2021 Carlier et al. https://creativecommons.org/licenses/by/4.0/ This content is distributed under the terms of the Creative Commons Attribution 4.0 International license.

### A hierarchical population genetic structure.

**FIG 2:**
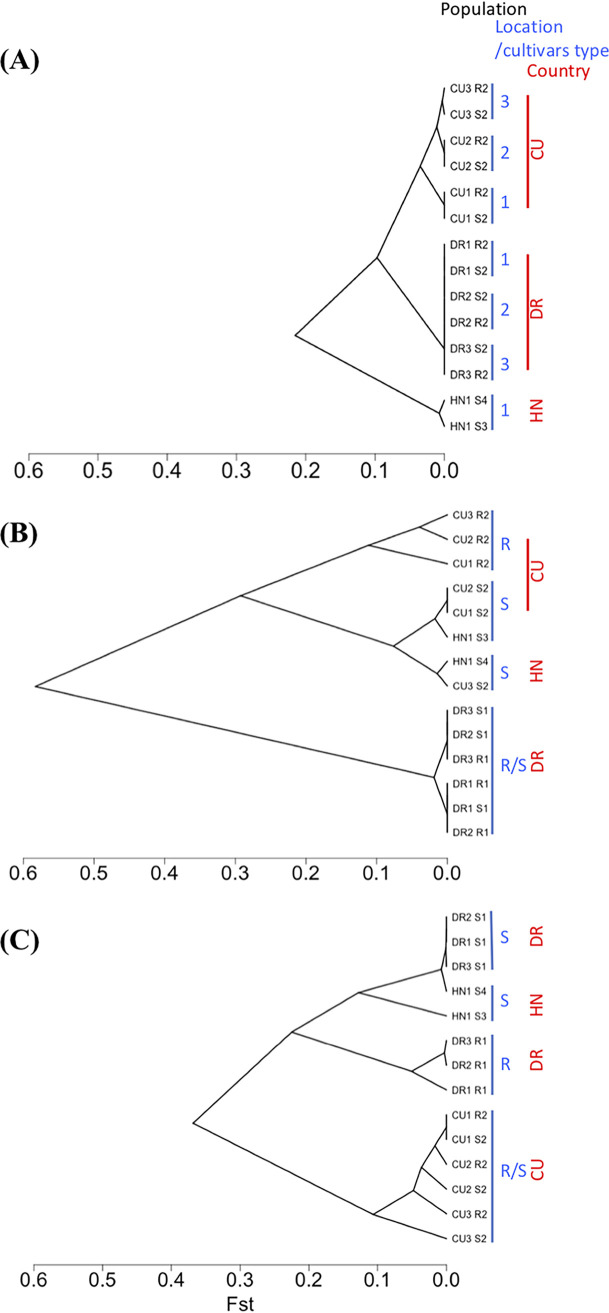
Dendrograms constructed from the *F_st_* between populations from Cuba (CU), the Dominican Republic (DR), and Honduras (HN) calculated from all SNPs along the genome (A) and SNPs within genomic regions having more evidence supporting selection footprints (classes I and II, Table 3) detected in Cuba (B) or in the Dominican Republic (C). R, resistant host; S, susceptible host. Each sample name indicates its country of origin (DR, CU, or HN), the code of the location of origin within countries (numbered 1, 2, or 3; Table 1 gives correspondences), and the code of the cultivar of origin (S1, susceptible cultivars ‘Macho’; S2, susceptible cultivars ‘Macho 3/4’; S3, susceptible cultivars ‘Grande Naine’; S4, susceptible cultivars ‘French sombre’; R1, resistant cultivars ‘FHIA21’; R2, resistant cultivars ‘FHIA18’).

The population genetic structure was first inferred from *F_st_* estimated from all SNPs (Fig. 2A and Table 2). A similar structure was obtained using the Bayesian hierarchical core model implemented in BayPass (Fig. S2). This structure was in line with the population history of *P. fijiensis* in the Latin America-Caribbean region previously inferred with 16 microsatellite markers (27)—from an initial introduction point in Honduras around 1972, the disease spread across the region through a series of serial population bottlenecks and was detected more recently in the Caribbean islands (from 1990). This history led to a hierarchical genetic structure as inferred from *F_st_* estimated from all SNPs (Fig. 2A and Table 2). Nucleotide diversity and Tajima’s D were estimated in each population (Table 2). Tajima’s D is a population genetic statistic that provides information on the allelic frequency spectrum and evolutionary forces acting on a DNA sequence (40). The negative Tajima’s D values obtained indicated an excess of low-frequency alleles, which is often interpreted as resulting from a population expansion after a recent bottleneck (41). On the whole core genome, nucleotide diversity was 1.4- to 10-fold lower in the Caribbean islands than in Honduras, and Tajima’s D was slightly positive for the Honduran populations and negative for all the DR and Cuban populations, thereby suggesting the existence of recent bottlenecks and population expansions in the Caribbean islands (Table 2). In DR and Cuba, the population structure reflected the geography (Fig. 2A): populations from the different locations were grouped together, and there was no evidence that populations from susceptible and resistant hosts were more related to each other. This means that any clear-cut differentiation between populations from different types of host (susceptible versus resistant) would likely be relatively insensitive to the population structure within countries and could reflect local adaptation.

**TABLE 2:** Ranges of *F_st_* estimated between populations within or between countries, median nucleotide diversity (π), and median Tajima’s D (D) estimated within populations on the core genome in each country

	*F_st_* (%)			π	D
	DR^*a*^	Cuba	Honduras		
DR	0.0	6.0/9.7	19.1/21.5	0.00020/0.00051	−2.057/−1.102
Cuba		0.0/3.9	6.2/16.4	0.00065/0.00203	−1.048/−0.092
Honduras			0.7	0.00269/0.00284	0.096/0.116

a DR, Dominican Republic.

10.1128/mBio.03129-20.2 FIG S2 Representation of the correlation plot (A) and a hierarchical clustering tree (B) derived from the matrix Ω of allelic frequencies estimated under the core model of BayPass with the study sanples from the Dominican Republic (DR), Cuba (CU), and Honduras (HN). Each sample name indicates its country of origin (DR, CU, or HN), the type of the cultivar of origin (R, population originating from a resistant host; S, population originating from a susceptible host), and the code of the location of origin within countries (1, 2, or 3; Table 1 gives correspondences). Download FIG S2, TIF file, 2.0 MB. Copyright © 2021 Carlier et al. 2021 Carlier et al. https://creativecommons.org/licenses/by/4.0/ This content is distributed under the terms of the Creative Commons Attribution 4.0 International license.

### Convergent selection footprints for different cultivars.

The detection of genomic regions exhibiting, in any paired population, strong divergence relative to the average divergence across the genome is evidence that in at least one of the populations these regions might have been involved in host selection and then adaptation. It could be considered that convergent adaptation has occurred if the same regions are found to have diverged in the multiple paired populations sampled (18, 19).

We sought to identify genomic regions putatively under convergent host selection between the locations sampled. To this end, as a first step, two different methods were run separately for paired populations from DR or Cuba since the cultivars sampled differed between the two countries. The Honduras samples collected only from other susceptible cultivars at a single location were not included in these analyses. As recommended by de Villemereuil et al. (42), we cross-checked our results using two single-marker methods with different power and sensitivity, i.e., a differentiation-based method (poolFreqDiff [43]) and a genotype-environment association method (BayPass [44]). The poolFreqDiff method tests for consistent allele frequency differences between population pairs across locations. Based on a quasibinomial generalized linear model (QGLM), this method performs better than the conventional Cochran-Mantel-Haenszel (CMH) test which may confuse heterogeneity and main effects (43). With the BayPass method, associations between SNPs and the cultivars of origin of the populations (resistant versus susceptible) were tested using three covariables. The first covariable (Cov-co) was qualitative and corresponded to the cultivars of origin. We cross-checked our results using two other quantitative covariables estimated in the common garden experiments previously published in the work of Dumartinet el al. (6). Such comparisons are considered efficient for deciphering the genetic basis of adaptive traits (45). These two quantitative covariables (Cov-dS for susceptible and Cov-dR for resistant cultivars) corresponded to the least-square means (LSMeans) of the diseased leaf area estimated for each population in the work of Dumartinet et al. (6) from a cross-inoculation experiment. Putative genomic regions under convergent host selection were then identified using the local score approach based on the *P* values estimated by the above analysis (46). This method accounts for linkage disequilibrium from pool-seq data combining single-marker tests. Genomic regions encompassing linked loci with a relatively low *P* value (leading to local scores that exceed a certain threshold) are identified using this method. The value of the ξ parameter for the local score formula affects the range of *P* values considered (only markers with *P* values under 10^−ξ^ will contribute to the score [46]). Variable values for the ξ parameter of 1, 2, and 3 were taken as recommended from simulations in a recent study (47).

**FIG 3:**
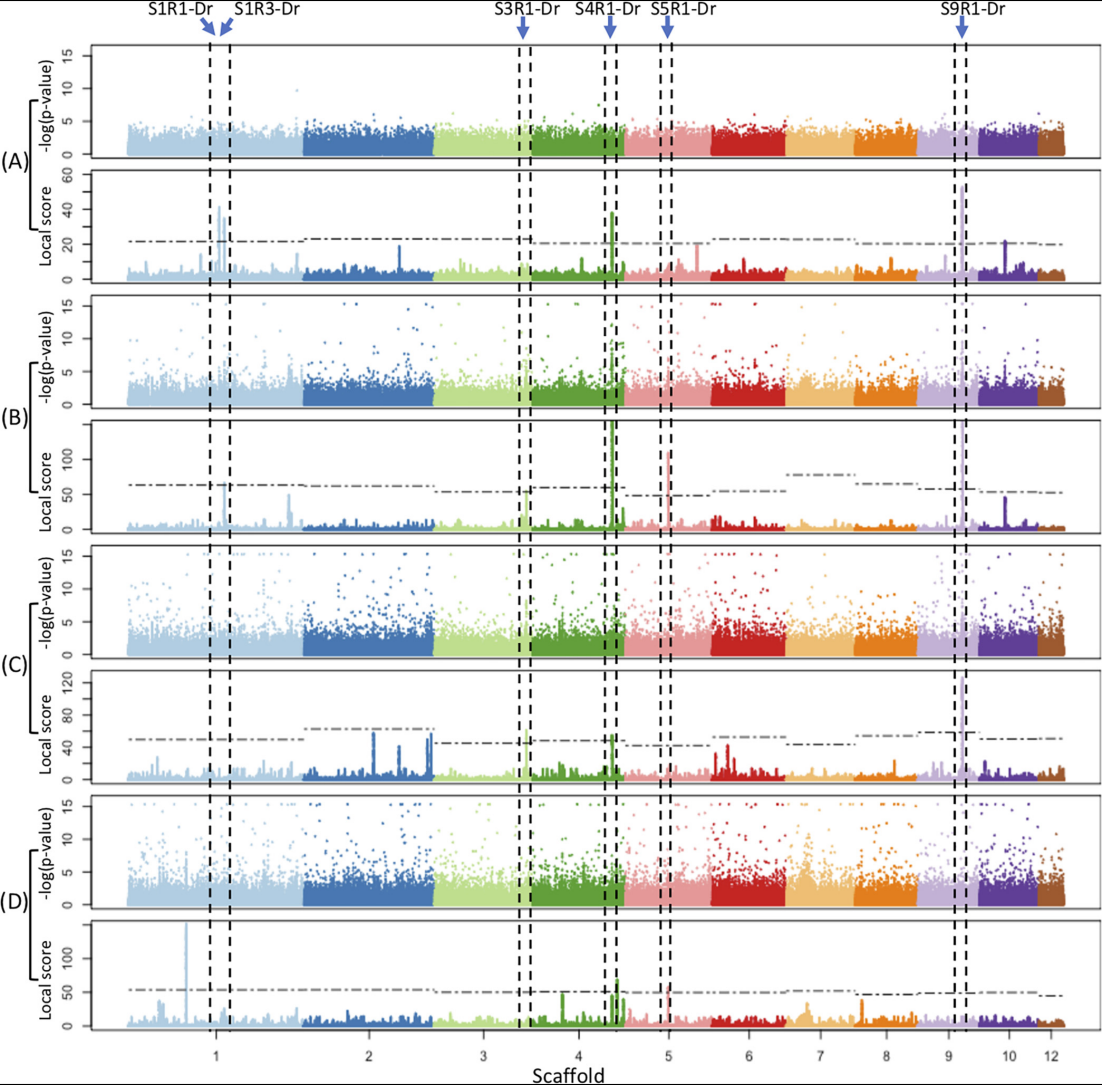
Manhattan plots showing selection footprints in the core genome of *P. fijiensis* detected between samples from different hosts collected from the Dominican Republic. Along the genome represented on the horizontal axis, the *P* values and local scores for each SNP are successively reported on the vertical axis from the test of allelic differentiation between populations sampled on resistant and susceptible cultivars using poolFreqDiff (A) or from the test of association using BayPass with the covariables Cov-co (B), Cov-dS (C), and Cov-dR (D). The horizontal dashed lines correspond to the chromosome-wide threshold α = 1% calculated for each scaffold. All genomic regions for which there was a concentration of SNPs above the threshold and for which there was more evidence supporting selection footprints (classes I and II, Table 3) are mentioned at the top.

According to the procedure presented above, eight and 16 genomic regions putatively under convergent host adaptation were detected in the DR and Cuban samples, respectively (Fig. 3, Table 3, and Fig. S3). Results obtained with a ξ value of 1 to 2 for poolFreqDiff and of 2 to 3 for BayPass were taken because the *P* values are globally lower with PoolfreqDiff. These regions were found in eight out of the 12 scaffolds analyzed, with a size ranging from around 0.6 to 70 kb. For each country, *F_st_* values were estimated in all of these regions and locations between population pairs isolated from susceptible and resistant cultivars.

**TABLE 3:**
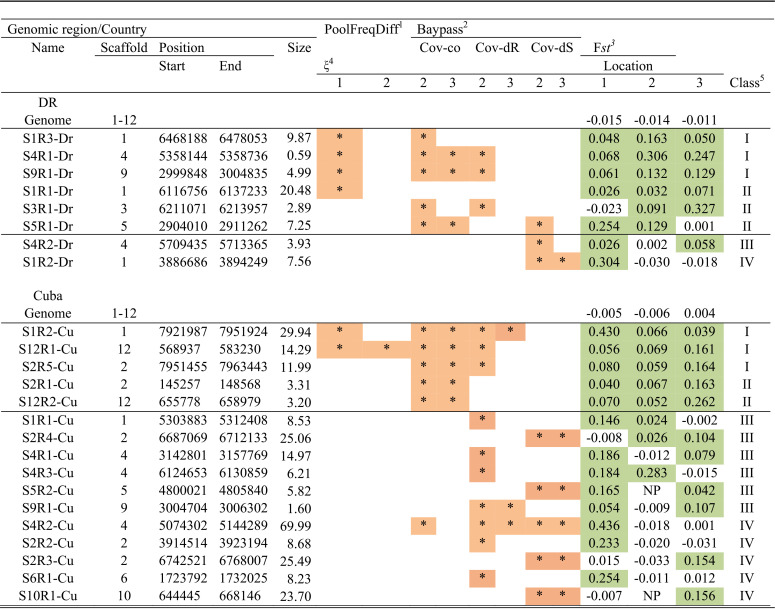
Genomic regions putatively under convergent host selection^*6*^

1 Genomic regions detected from poolFreqDiff.

2 Genomic regions detected from BayPass with Cov-co, Cov-dS, or Cov-dR as covariables.

3 *F_st_*, estimate between population pairs.

4 Value of the ξ parameter for the local score formula.

5 Genomic regions were classified in four selection-support classes (Table 4).

6 Asterisks highlighted in salmon pink indicate the methods from which the genomic regions were detected with a 1% significance threshold. The *F_st_* values highlighted in green were considered significant with a 5% significant threshold. Each region name indicates its scaffold number after the letter S (for scaffold), the region number after the letter R (for region), and the country where it was detected (DR for Dominican Republic or CU for Cuba).

10.1128/mBio.03129-20.3 FIG S3 Manhattan plots showing selection footprints in the *P. fijiensis* core genome detected between samples from different hosts collected in Cuba. Along the genome represented on the horizontal axis, the *P* values and local scores for each SNP are successively reported on the vertical axis from the test of allelic differentiation between populations sampled on resistant and susceptible cultivars using PoolFreqDiff (A) or from the test of association using BayPass with the covariables Cov-co (B), Cov-dS (C), and Cov-dR (D). The horizontal dashed lines correspond to the chromosome-wide threshold α = 1% calculated for each scaffold. All genomic regions for which there was a concentration of SNPs above the threshold and for which there was more evidence supporting selection footprints (classes I and II, Table 3) are mentioned at the top. Download FIG S3, TIF file, 2.5 MB. Copyright © 2021 Carlier et al. 2021 Carlier et al. https://creativecommons.org/licenses/by/4.0/ This content is distributed under the terms of the Creative Commons Attribution 4.0 International license.

Considering all the data, the regions were classified in four categories starting from those having more evidence supporting convergent adaptation (Table 4). For category I, regions were detected using the results from both the poolFreqDiff and BayPass methods combined, or only from BayPass, but using two covariables, and *F_st_* was significant for all three locations sampled. Three regions per country were in this category. For category II, regions were detected with poolFreqDiff or BayPass with only one covariable, and *F_st_* was significant in all three locations sampled. Category II also included regions detected using BayPass with two covariables, and *F_st_* was significant for two of the three locations sampled. Three and two regions were in this category in DR and Cuba, respectively. For category III, regions were detected with BayPass with only one covariable and *F_st_* was significant for two of the three locations sampled. One and six regions were in this category in DR and Cuba, respectively. Finally, for category IV, regions were detected with BayPass with only one covariable (except for S4R2-Cu detected with the three), and *F_st_* was significant for only one of the three locations sampled. One and five regions were in this category in DR and Cuba, respectively. Using BayPass with quantitative covariables, some regions were detected with Cov-dS (related to susceptible cultivars) or with Cov-dR (related to resistant cultivars), suggesting host specificity. Note finally that some regions were detected using poolFreqDiff and/or BayPass with Cov-co (corresponding to the cultivars of origin) and thus might be involved in a trait other than that measured in the cross-inoculation experiment but also showing some host specificity.

**TABLE 4:** Decision criteria used to quantify the strength of evidence in favor of convergent selection between population pairs collected from different cultivars across locations^*d*^

Class	Support	PoolfreqDiff	BayPass		Significant *F_st_*		
			2 cov.^*a*^	1 cov.^*b*^	3 locations	2 locations	1 location
I	Very strong	*	*		*		
		*		*	*		
			*		*		
II	Strong	*			*		
				*	*		
			*			*	
III	Moderate			*		*	
IV	Weak		(*)^*c*^	(*)^*c*^			*

a Genomic regions detected using Cov-co and either Cov-dS or Cov-dR as covariables (cov.).

b Genomic regions detected using Cov-dS or Cov-dR as covariables.

c Genomic regions detected using one and/or two covariables.

d Asterisks indicate the methods from which the genomic regions were detected with a 1% significance threshold (poolFreqDiff, BayPass).

Only class I and II regions, which had the most evidence of convergent adaptation between locations, were kept for further characterization. To help visualize these convergences, we calculated pairwise *F_st_* values from SNPs only within the detected class I and II regions in each country and constructed a dendrogram (Fig. 2B and C). When considering the detected regions within one country, here populations were first grouped according to host origins and not according to their location in the corresponding country.

### Analysis of the AFS.

**FIG 4:**
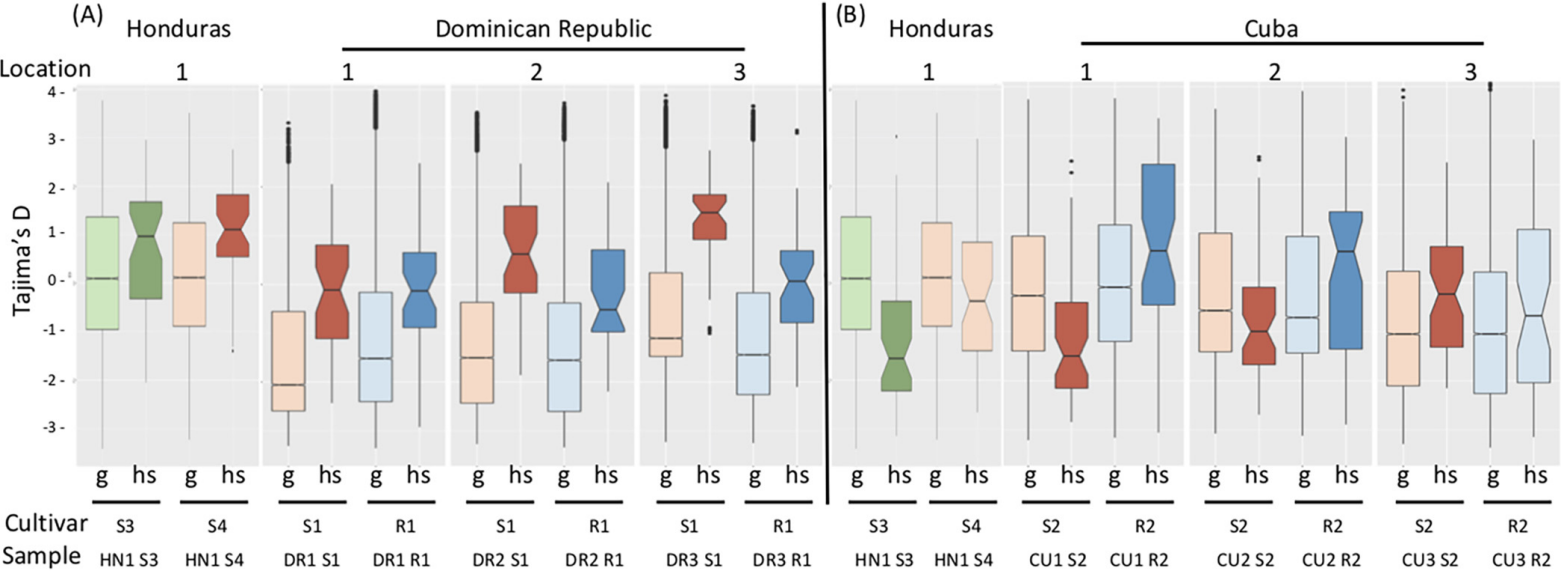
Boxplot of Tajima’s D estimates in the Dominican Republic (A) and Cuba (B) from different locations and cultivars. Estimates for genomic regions putatively under host selection (hs) significantly different from the estimate for the whole genome (g) at the 5% level using the Mann-Whitney U-test are indicated by darker colors. Each sample name indicates its country of origin (DR, Dominican Republic; CU, Cuba; HN, Honduras), the code of the location of origin within countries (numbered 1, 2, or 3; Table 1 gives correspondences), and the code of the cultivar of origin (S1, susceptible cultivars ‘Macho’; S2, susceptible cultivars ‘Macho 3/4’; S3, susceptible cultivars ‘Grande Naine’; S4, susceptible cultivars ‘French sombre’; R1, resistant cultivars ‘FHIA21’; R2, resistant cultivars ‘FHIA18’).

Allelic frequency spectrum (AFS) was analyzed in each population in the whole core genome and the class I and II regions pooled using Tajima’s D estimates on 1-kb nonoverlapping windows (Fig. 4). An increase or decrease in Tajima’s D in the putative selected regions relative to the whole core genome would indicate an increase of intermediate-frequency alleles or low-frequency alleles, respectively (41). From the genomic regions detected in DR, a significant increase in Tajima’s D was detected in all samples from Honduras and DR, regardless of the cultivars. However, the extent of the difference between Tajima’s D estimated from the whole genome and that from the selected regions was greater for the DR samples than for those from Honduras. From the genomic regions detected in Cuba, a significant increase in Tajima’s D was again detected for one cultivar in the Honduran samples and in only three Cuban samples, corresponding to the resistant cultivars at locations 1 and 2 and to susceptible cultivars at location 3. A significant lower mean Tajima’s D was detected in two other samples from the susceptible cultivars at locations 1 and 2. Thus, in two of the three locations in Cuba, the mean Tajima’s D for the selected regions relative to the whole changed in opposite directions between susceptible and resistant cultivars. These observations suggested that the allele frequency spectra had changed in all the putative selected regions relative to the whole genome for most of the populations, regardless of their host origin.

### Some genes in candidate genomic regions might be involved in host-pathogen interactions.

From two to 11 genes were identified in each class I and II region, with 47 overall (Table 5). All of the information available to date for these genes is provided in Table S1. No GO terms were significantly overrepresented within the 47 genes. Furthermore, none of the detected genes coded for SSPs, i.e., the putative effectors of plant-pathogen interactions. However, as molecules other than SSPs secreted by pathogens could be considered potential effectors (10), we assessed whether or not the identified genes belonged to the set of genes having a signal peptide. Four of these genes were detected in three different regions, and two of them were also described in the *in vitro* secretome of *P. fijiensis* (48). One of these genes (ID 55415), detected in region S1R2-Cu, coded for a putative glucoside hydrolase differentially secreted *in vitro* by a *P. fijiensis* isolate having a higher pathogenicity than another one (48). This gene also had a homologue in the pathogen-host interaction database (PHI-base [49]) which codes for an effector of the Blumeria graminis f. sp. *hordei* fungus found to be involved in a reduction in pathogenicity in a silencing experiment (67). Two other genes with a signal peptide (regions S1R1-Dr and S2R1-Cu) had unknown functions and were potential effectors. The latter gene with a signal peptide was significantly more expressed in infected leaf tissue than in a culture medium in the transcriptome analysis published by Noar and Daub (50) and had homology with galactosyltransferase. Overall, eight genes distributed in five genomic regions were homologous to a fungal gene of the PHI-base (49) that has been demonstrated to have an effect on quantitative pathogenicity (phenotype “reduced virulence,” or “loss of pathogenicity,” or “hypervirulence”). Three of these genes were involved in gene regulation at the transcriptional level (with a lysine histone methyltransferase and a transcription factor) or at the posttranslational level (with a protein kinase).

**TABLE 5:** Summary of the gene annotation results in the genomic regions putatively under convergent host selection^*g*^

Genomic region	Scaffold^*a*^	Size (kb)	Annotation						Protein ID^*f*^
			No. of genes	Sp^*b*^	Secr.^*c*^	Trans.^*d*^	BLAST^*e*^	Putative function/homologue	
S1R3-Dr	1	9.87	4				3	Kinase of Fusarium graminearum (81)	213358
								Beta-oxidase of Magnaporthe oryzae (82)	149244
								Multidrug resistance; protection from oxidative stress from Magnaporthe oryzae (83)	2013360
S4R1-Dr	4	0.59	0						
S9R1-Dr	9	4.99	0						
S1R1-Dr	1	20.48	11	1	1			Glycoside hydrolases	46143
				1				Unknown	170196
S3R1-Dr	3	2.89	3	1		1		Galactosyltransferase	153261
S5R1-Dr	5	7.25	2				1	Transcription factor of Magnaporthe oryzae (84)	203908
S1R2-Cu	1	29.94	12	1	1		1	Glycoside hydrolases. Effector protein of Blumeria graminis (67)	55415
							2	Lysine histone methyltransferase of Fusarium verticillioides (85)	149458
								Superoxide dismutase of Puccinia striiformis, reduced virulence (86)	112476
S12R1-Cu	12	14.29	5	1				Unknown	200624
							1	Short-chain dehydrogenase of Magnaporthe oryzae (87)	83266
S2R5-Cu	2	11.99	6						
S2R1-Cu	2	3.31	2			1		Unknown	210184
S12R2-Cu	12	3.20	2						
Total			47	5	2	2	8		

a Scaffold where genomic regions are located.

b Genes having a predicted peptide signal.

c Genes of *in vitro* secretome of *P. fijiensis* differentially expressed in virulent isolates compared to avirulent isolates (48).

d Genes with lower or higher expression in infected leaf tissue compared to culture medium (50).

e BLAST search results from the Pathogen-Host Interactions database (PHI-base [49]); best hits with plant-pathogenic fungus genes having an effect on pathogenicity.

f JGI protein IDs from the *P. fijiensis* reference genome (https://genome.jgi.doe.gov/Mycfi2/Mycfi2.home.html [28]).

g Gray areas indicate characteristics of the same genes.

10.1128/mBio.03129-20.4 TABLE S1 Annotation of genes in genomic regions putatively under convergent host adaptation. Download Table S1, XLSX file, 0.02 MB. Copyright © 2021 Carlier et al. 2021 Carlier et al. https://creativecommons.org/licenses/by/4.0/ This content is distributed under the terms of the Creative Commons Attribution 4.0 International license.

## DISCUSSION

In this study, we used a pool-seq approach to compare samples of the fungus *P. fijiensis* that had evolved on banana cultivars with different levels of quantitative resistance in the Dominican Republic and Cuba. The results revealed convergent selection signatures for the cultivars across the sampled locations in each country in some genomic regions that included candidate genes involved in host adaptation and quantitative pathogenicity, thereby supporting the existence of convergent and oligogenic adaptation to quantitative resistance.

Local adaptation to quantitative resistance has already been described in fungi (51–55) and recently in *P. fijiensis* (6). However, the samples in these plant inoculation-based studies came from a single location, or the number of isolates sampled from different locations was small. Convergent adaptation was sometimes suggested but not clearly demonstrated, and the genetic basis of adaptation to quantitative resistance was not addressed. Our study involved a population genomics approach, and the findings strongly supported convergent selection signatures between locations for different cultivars within each study country and thus convergent host adaptation. Five to six genomic regions considered strongly or very strongly supporting convergent adaptation were detected in the two countries (the class I and II regions outlined in Results [Fig. 2 and 3 and Fig. S2; Tables 3 and 4]). There was also moderate evidence of convergence in one to six more regions in the two countries (class III regions). Different sets of genes could be found to be involved in adaptation between locations, and greater support for convergence might be detected in more genomic regions if a larger number of locations were to be studied. Convergent adaptation between the two study countries was not observed since there was no overlap between the two sets of genomic regions detected in both of them. However, the cultivars sampled in DR and Cuba differed. The resistant cultivars (FHIA18 and FHIA21) shared the same resistant parent, but the latter might have some level of heterozygosity which could lead to segregation for this trait in crosses. Furthermore, the other parents were different, and some of their genes transmitted in new hybrids might also play a role in their quantitative resistance.

Considering the regions most supported for convergent adaptation signatures (classes I and II), the results of this study implied that quantitative pathogenicity and adaptation to QR in *P. fijiensis* is at least oligogenic. This is in line with the findings of a recent comprehensive QTL mapping analysis conducted on *Z. tritici* (13), a fungus related to *P. fijiensis*, belonging to the same family (Mycosphaerellaceae within the Dothideomycetes class) and having a similar biology (56), for which a complex genetic architecture of quantitative pathogenicity was documented. The evolution of quantitative pathogenicity can potentially be determined by several quantitative plant-pathogen interaction traits, each depending on the expression of several genes in the pathogen. On the plant side, recent comprehensive studies showed that quantitative resistance depends on several dozen genes (7). Complementary results obtained on the quantitative pathogenicity of some fungi suggest that molecular interactions between plants and pathogens in quantitative disease development may be complex and involve several genes on both sides, in contrast with ETR.

A change in the allele frequency spectrum, in comparison with the whole genome, was detected in genomic regions for which there was more evidence of host selection (classes I and I [Tables 3 and 4]) in most of the populations analyzed and regardless of the cultivars of origin (Fig. 4). These observations supported the assumption that, in comparison with the rest of the genome, evolutionary forces interact differently on loci in these regions, which thus might be involved in host-pathogen interactions in all of the study cultivars. It is worth noting that susceptible cultivars, which can be considered to have low quantitative resistance, may also interact with and exert some constraints on the pathogen. The estimated Tajima’s D statistic comparison findings were not always compatible with what one might expect according to the theory of classical so-called hard selective sweeps (i.e., lower values in the genomic regions under selection with an excess of low-frequency variants [29, 57]). Instead, in most populations we observed an increase in Tajima’s D values in regions putatively under host selection, suggesting an increase in alleles with intermediate frequencies. This pattern is expected when a selective sweep is incomplete, or ongoing following the introduction of new selection pressure (58, 59), and when it is soft (60). In accordance with this observation, the dissemination of the resistant cultivars (about 15 years ago) and the introduction of *P. fijiensis* in both of the study countries (20 to 30 years ago) were very recent events (25). Thus, *P. fijiensis* has been adapting to the cultivars for more than a decade. Furthermore, the interaction of demographic events with selection can impact the Tajima’s D values. An abundance of regions with positive Tajima’s D values was recently observed in a population genomics study on Drosophila simulans, a species that has also recently colonized a new geographical region (North America). Moreover, simulations on different demographic scenarios showed that positive Tajima’s D values are expected, on average, when a selective effect and a recent population contraction occur concomitantly (61). This scenario seems to be close to that followed by *P. fijiensis* populations in the Caribbean since they faced new hosts during their expansion after initial recent bottlenecks. However, it has not been possible to conduct simulations to test the effects of *P. fijiensis* population-history scenarios on statistics such as Tajima’s D since reliable estimates of demographic and genetic parameters are currently not available for this species and sequencing of individuals would be required (62).

In the presence of specific interactions and restricted gene flow, divergent selection between habitats (hosts here) could lead to local adaptation (63, 64). As the durability of a given resistance may depend on its specificity level, this aspect needs to be more documented with regard to QR (3, 5). The existence in some genomic regions of genetic differentiation between population pairs and of significant changes in Tajima's D values in most populations suggested that divergent selection between cultivar types (susceptible versus resistant) has occurred. This implies that all of the cultivars might be impacted by some specific host-pathogen interactions. The existence of such interactions has recently been suggested in plant inoculation studies with *P. fijiensis* but only on resistant cultivars (6). Specific interactions have been detected, or not, in other fungi via inoculations (51–55). The detection, or not, of specificities may depend on the underlying genes involved in the interactions but also on the statistical power of the experimental design, given that the sample size that can be analyzed in inoculation assays is very limited. As we were able to analyze a larger number of samples, our population genomics approach may be more powerful for detecting specificity on all cultivars, including those that are supposedly susceptible and which may be considered to have low quantitative resistance.

Combining the annotation results for the genes identified in the candidate genomic regions using the *P. fijiensis* reference genome with the findings of functional studies on this and other pathogens indicated that some of the genes might be involved in host-pathogen interactions. The genes detected in the most supported genomic regions (classes I and II) are presented in Table 5 and in Table S1 in the supplemental material. This list could be first considered for future experimental validation. No genes were detected in two out of 11 of the identified genomic regions. However, in the populations analyzed, some genes may exist in these regions that are not present in the reference genome used for the mapping step. A recent study of 19 *de novo*-assembled genomes using long-range sequencing technology with the related fungus Zymoseptoria tritici showed that conserved orthogroups accounted for only about 60% of the species pangenome (65). Furthermore, the 40% left represented an accessory pangenome that varied between isolates and was enriched in pathogenesis-related functions including more than 60% of the predicted effectors. *De novo* assembly and annotation of some *P. fijiensis* genomes from the study populations will certainly improve the identification of the genes involved. None of the 47 genes detected corresponded to the putative effector SSP identified *in silico* in the work of Arango Isaza et al. (28). However, it has been proposed that effectors should be defined as any microbial secreted molecule that contributes to niche colonization (66). Thus, all secreted proteins are potential effectors, and via the population genomic approach we used in this study, we detected three genes encoding non-SSP proteins yet which presented a peptide signal. One of them seemed to be the best candidate of this study. This gene was detected in the S1R2-Cu genomic region and corresponded to glycoside hydrolase, which is secreted to a greater extent *in vitro* in a highly pathogenic isolate of *P. fijiensis* (48) and has high homology with a gene and a putative effector involved in the pathogenicity of the fungus *Blumeria graminis* f. sp. *hordei* (67). However, although it was secreted, it was instead considered to be a candidate morphogenetic factor (but essential for pathogenesis) rather than an effector in the strict sense (67). Homology with genes involved in pathogenicity was found in most of the other genomic regions (but not coding for putative effectors), and genes detected in three genomic regions were homologous with genes involved in gene regulation. We therefore did not obtain any clear evidence that QR erosion of banana cultivars results from changes in effectors. Complementary to functional studies, our population genomics approach might enable the identification of genes other than *in silico* predicted effectors involved in both quantitative pathogenicity and erosion of quantitative resistance in plants.

This study showed that pool-seq, combined with a paired population sampling design and recent analysis methods, is an efficient approach for detecting convergent host adaptation in plant-pathogenic fungi while also enhancing the identification of candidate genes involved in such adaptation. The main results suggested that the genetic basis of fungal adaptation to QR is at least oligogenic and highlighted the existence of specific host-pathogen interactions for this kind of resistance. However, even in the presence of QR specificity, tradeoffs in the adaptation of pathogen populations to the different host genotypes might exist and help define durable disease deployment strategies to hamper pathogen adaptation. To that end, it would now be essential to study this genetic basis of fungal adaptation to QR in a broader range of quantitative-resistant genotypes. In *P. fijiensis*, such studies could be conducted on a wide range of diploid bananas that have already been described as featuring QR that could potentially serve as parents in breeding programs (25).

## MATERIALS AND METHODS

### Pool-seq and SNP calling.

Mycelium cultures initiated by single ascospores isolated from necrotic lesions bearing perithecia were identified as belonging to *P. fijiensis* and stored as described in reference 68. All isolates were thus the product of sexual reproduction and corresponded to different individuals. They were genotyped using 16 microsatellites markers in the previous study of Dumartinet et al. (6), and genotypic diversity was close to the maximum value possible with no significant linkage disequilibrium in each population. In the present study, genomic analysis was based on 32 to 58 (mean = 42.25) isolates in DR and Cuba and 27 to 30 isolates in Honduras (Table 1). Isolates were pooled after DNA extraction. As suggested in a recent theoretical study (36), this approach appeared to be the best way to reduce variation in the pooling step of our study. Mycelia from each isolate were grown on solid medium (300 ml V8, 3 g CaCO_3_, 20 g/liter agar, pH 6) for 2 weeks at 25°C, dried for 2 days at 55°C, and ground. Genomic DNA was extracted as detailed in reference 69. Equimolar amounts of DNA from 27 to 58 isolates were pooled for each sampled population (Table 1). Overall, 17 pools were sequenced using paired-end Illumina sequencing at the Genome Quebec Innovation Centre at McGill University on the GAII platform. Samples were (6 per lane) sequenced with a 100-bp read length and a targeted depth of 80×. The pool of population DR3 R1 was sequenced four times with four independent sequencing libraries to help filter sequencing errors after SNP calling.

Genomic reads from fastq files were mapped against the *P. fijiensis* reference genome (https://genome.jgi.doe.gov/Mycfi2/Mycfi2.home.html [28]) using Stampy aligners 1.0.13 with default settings (70) and with a minimum mapping quality score of 30. Sites around indels were realigned using the Genome Analysis Toolkit (GATK) v1.05777 indel realigner (71). Coverage was analyzed from the generated BAM file using the Genocov program from Bedtools v.2.27.0 (72). Based on the coverage distributions, a minimum coverage of 20 and a maximum coverage of 150 were used as thresholds for SNP identification to correct for potential errors from repetitive sequences. Considering these thresholds, the mean coverage was between 55 and 85× and then was always higher than the number of individuals, as recommended in reference 36. The proportion of the genome represented was between 57 and 62% across all samples, suggesting that most of the repetitive sequences were eliminated, as these sequences amounted to around 40% of the *P. fijiensis* genome (28). According to the PoPoolation2 pipeline for pool-seq data (73), a synchronized file was created via SNP calling using SAMtools mpileup (74). The popsync2pooldata function of the R package Poolfstat (75) was used to filter biallelic SNPs with coverage of between 20 and 150× in each sample. To set up filtering in this study, the four sequencing replicates of the same pool (DR3 R2) were first used together to test values for the minimum read count per base (min.rc) with 1 ≤ min.rc ≤ 4 and for the minimum allele frequency (MAF) with 0 ≤ MAF ≤ 0.05. Since the DNA pool sequenced was the same among replicates, SNPs detected with a given set of parameters were obviously false and resulted from sequencing errors. The number of SNPs decreased quickly when both parameters increased and became independent of min.rc when MAF = 0.03, suggesting that most sequencing errors were filtered (see Fig. S1 in the supplemental material). A min.rc = 3 and a MAF = 0.03 were then adopted to filter all the samples.

### Population genetic structure.

We described the population structure by first estimating the pairwise *F_st_* on all loci between populations using the R package Poolfstat (75) and drew up dendrograms using the standard R functions hclust and plot. We also calculated the covariance matrix Ω of the population allele frequencies by running the core model of the BayPass program (44).

### Genome scan for convergent selection footprints.

In a PoolFreqDiff analysis, we rescaled all the allele counts to the effective sample size (*n*_eff_), as recommended by the authors (43). A *P* value for each SNP was calculated from a G-test of consistency between population pairs across locations. In BayPass analysis, the qualitative covariable (Cov-co) corresponding to the cultivars of origin was coded as 1 for resistant and −1 for susceptible, as performed in the work of Eoche-Bosy et al. (76). The quantitative covariables Cov-dS and Cov-dR were estimated in the work of Dumartinet et al. (6) using a subsample of 16 to 32 isolates drawn from the same samples analyzed in the present study. An empirical Bayesian *P* value was calculated for each SNP for an association with the covariables tested using the standard covariate model. Three independent runs were conducted for all the BayPass analyses and gave very close results. For the local score approach, as the *P* value distributions in the above analysis were nonuniform (chi-squared test, *P* < 0.0001), we used the resampling procedure proposed in the work of Fariello et al. (46) to approximate a null distribution and defined a chromosome-wide significance threshold. We opted for a 1% threshold based on the variable value for the ξ parameter. This procedure is jointly sensitive to the correlation between loci and the chromosome size. Scaffolds that definitely corresponded to dispensable (B) chromosomes (77) in *P. fijiensis* generally showed higher correlations of single-marker *P* values, which may have been the result of less reliable mapping due to greater variability between the reference genome and the sequenced pools. B chromosomes are also smaller in size (28). Thus, we ran the local score approach and all the other analyses while considering only the 12 scaffolds corresponding to the core genome from a synteny analysis (28). This core set encompassed almost 87% of the genome and more than 96% of the predicted genes.

### Genetic differentiation within putative selected regions.

The existence of convergent host selection between locations in the genomic regions detected with the above two methods was also addressed using an independent *F_st_* outlier approach between population pairs at each location. *F_st_* values were estimated for all the locations sampled and the genomic regions detected using the estimator developed for pool-seq data and implemented in the R package Poolfstat (75). As *P. fijiensis* is not a model organism, estimates of demographic models and genetic parameters are not available to draw a null distribution of *F_st_*. The pool-seq data provided in this study were not ideal for fitting a demographic model of *P. fijiensis* in the geographical region studied, which would have required genome sequencing of separate individuals (62). Furthermore, false positives can be generated with this approach if the modeled and true demography do not match (29). We hence preferred an empirical approach rather than a model-based approach to define the null distribution of *F_st_*, since it can represent simple and transparent data treatments (e.g., see reference 78). For each region and population pair, the null *F_st_* distribution was defined from *F_st_* estimates for SNPs in all the nonoverlapping windows along the entire genome having the same size as the considered region and with a minimum number of 10 SNPs. The number of windows taken for each region was over 2,000, except for the longest one (Cu4R1 of about 60 kb in size) for which the number was 900. *F_st_* values were considered significant when they fell in the 5% upper tail of the *F_st_* distribution. Dendrograms based on *F_st_* values estimated on subsets of genomic regions were constructed as described above for the population genetic structure.

### Within-population statistics.

Nucleotide diversity π and Tajima’s D were evaluated using the unbiased estimators implemented in PoPoolation (79) using 1-kb nonoverlapping windows. The difference between the median Tajima’s D values estimated on the whole genome and those on genomic regions putatively under convergent adaptation was tested using a Mann-Whitney U-test.

### Analysis of loci in candidate genomic regions.

The annotated *P. fijiensis* reference genome was used to define the list of genes included within the genomic regions putatively under host selection. The annotation (GO terms, KOG terms, presence of a peptide signal) for these genes was retrieved from GFF3 files on the JGI website (https://genome.jgi.doe.gov/Mycfi2/Mycfi2.home.html). Enrichment for GO terms was tested using the Gowinda program (78). We also tested whether these genes corresponded to *in silico*-defined SSPs (28) or to proteins secreted *in vitro* and *in planta* by comparing isolates with different pathogenicity levels (48) or genes expressed during infection in a transcriptome analysis (50). Protein sequences were subjected to a BLAST (80) search in the pathogen-host interactions database (PHI-base), which currently contains around 6,000 genes proven to affect the outcome of host-pathogen interactions (49). Almost 70% of the host species in this database belong to plants. For each BLAST search, we kept the gene in the PHI-base that had the lowest bitscore (and E value < 1 × 10^−9^) and an effect on quantitative pathogenicity (i.e., phenotype “loss of pathogenicity.” “reduced virulence,” or “increased virulence”).
