# Supplementary figures and images for: Convergent Adaptation to Quantitative Host Resistance in a Major Plant Pathogen
Source: mBio. 2021 Feb 23;12(1):e03129-20. doi: 10.1128/mBio.03129-20 (PMC8545102; doi:10.1128/mBio.03129-20)

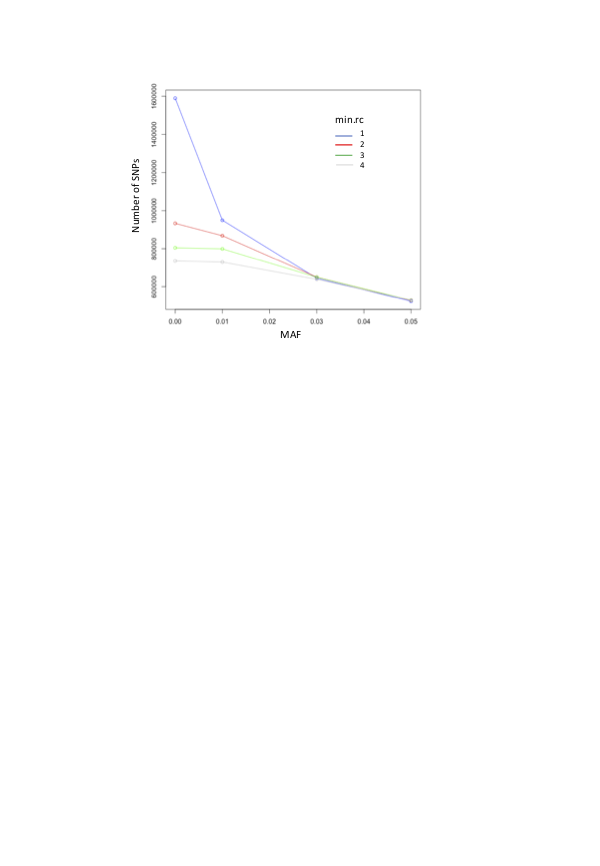

Supplement: FIG S1 [file mbio.03129-20-sf001.tif]

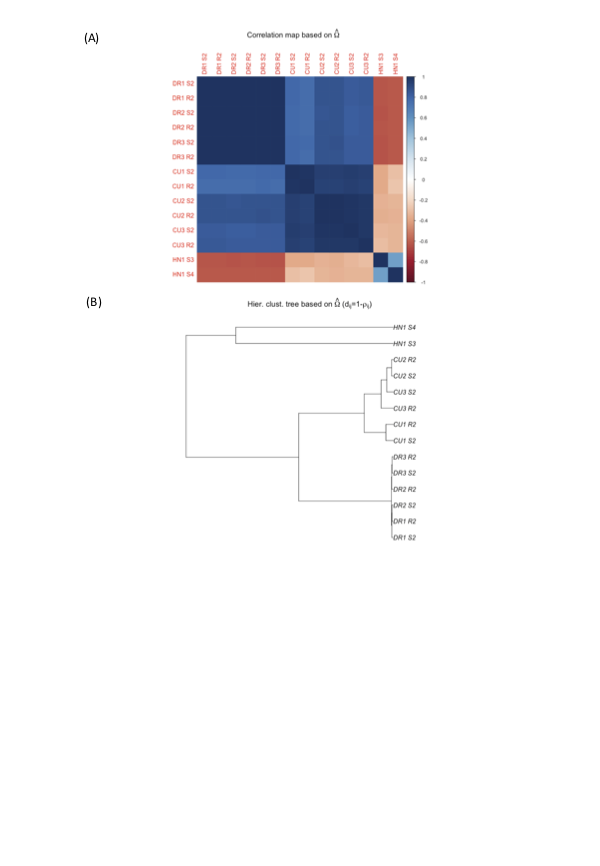

Supplement: FIG S2 [file mbio.03129-20-sf002.tif]

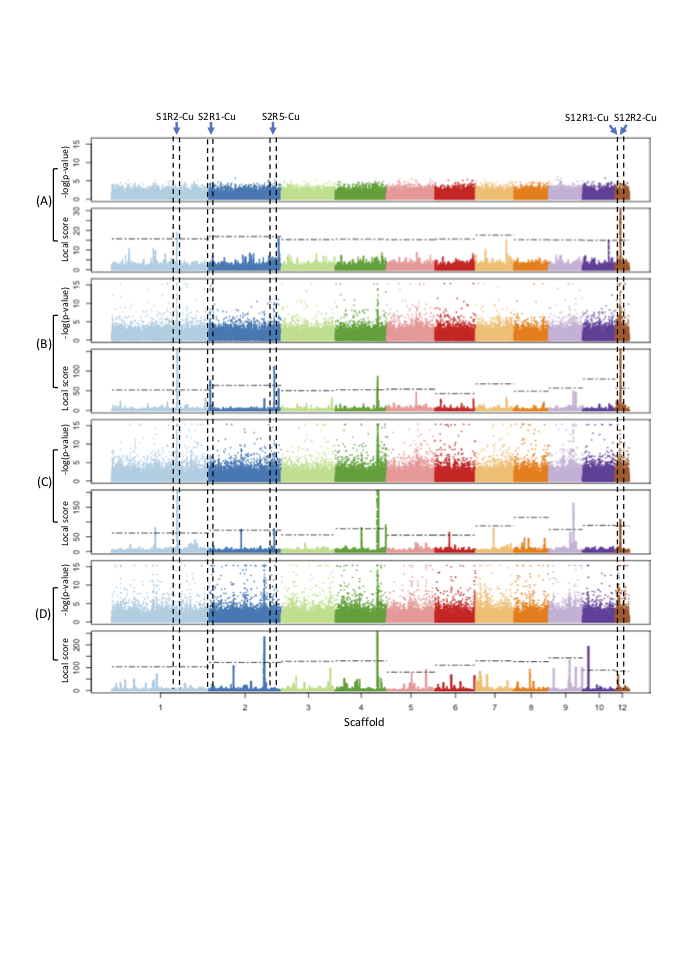

Supplement: FIG S3 [file mbio.03129-20-sf003.tif]
